# Increasing the Fungicidal Action of Amphotericin B by Inhibiting the Nitric Oxide-Dependent Tolerance Pathway

**DOI:** 10.1155/2017/4064628

**Published:** 2017-10-10

**Authors:** Kim Vriens, Phalguni Tewari Kumar, Caroline Struyfs, Tanne L. Cools, Pieter Spincemaille, Tadej Kokalj, Belém Sampaio-Marques, Paula Ludovico, Jeroen Lammertyn, Bruno P. A. Cammue, Karin Thevissen

**Affiliations:** ^1^Centre of Microbial and Plant Genetics, KU Leuven, 3001 Heverlee, Belgium; ^2^BIOSYST-MEBIOS, KU Leuven, 3001 Heverlee, Belgium; ^3^VIB Department of Plant Systems Biology, 9052 Ghent, Belgium; ^4^Department of Laboratory Medicine, University Hospitals Leuven, Herestraat 49, 3000 Leuven, Belgium; ^5^Life and Health Sciences Research Institute (ICVS), School of Health Sciences, University of Minho, Braga, Portugal; ^6^ICVS/3B's-PT Government Associate Laboratory, Braga/Guimarães, Portugal

## Abstract

Amphotericin B (AmB) induces oxidative and nitrosative stresses, characterized by production of reactive oxygen and nitrogen species, in fungi. Yet, how these toxic species contribute to AmB-induced fungal cell death is unclear. We investigated the role of superoxide and nitric oxide radicals in AmB's fungicidal activity in *Saccharomyces cerevisiae,* using a digital microfluidic platform, which enabled monitoring individual cells at a spatiotemporal resolution, and plating assays. The nitric oxide synthase inhibitor L-NAME was used to interfere with nitric oxide radical production. L-NAME increased and accelerated AmB-induced accumulation of superoxide radicals, membrane permeabilization, and loss of proliferative capacity in *S. cerevisiae*. In contrast, the nitric oxide donor S-nitrosoglutathione inhibited AmB's action. Hence, superoxide radicals were important for AmB's fungicidal action, whereas nitric oxide radicals mediated tolerance towards AmB. Finally, also the human pathogens *Candida albicans* and *Candida glabrata* were more susceptible to AmB in the presence of L-NAME, pointing to the potential of AmB-L-NAME combination therapy to treat fungal infections.

## 1. Introduction

Pathogenic fungi, including *Candida albicans* and *Candida glabrata*, encounter diverse environmental stresses when colonizing human tissues. During the infection process, they are exposed to potent reactive oxygen and nitrogen species (ROS and RNS, resp.), including nitric oxide radical (NO^•^), peroxynitrite (ONOO^−^), superoxide anion radical (O_2_^−•^), and hydroxyl radical (^•^OH), generated by the respiratory burst of phagocytic cells [1–3]. ROS and RNS cause damage to DNA, proteins, and lipids and are toxic to most fungi [4, 5]. In contrast to most nonpathogenic fungi, fungal pathogens such as several *Candida* species have developed responses to neutralize these toxic radicals and repair the potential molecular damage [6]. In this respect, various proteins that protect the fungus from oxidative and nitrosative stresses have been identified and include signalling proteins, transcription factors, and a variety of other enzymes such as catalases, superoxide dismutases, peroxidases, and nitric oxide dioxygenase [1, 7]. Hence, antifungals (or combinations thereof) inducing an excess ROS and/or RNS in a pathogenic fungus that cannot be neutralized by its endogenous protection mechanisms are of great interest [8].

Many antifungal agents are reported to induce oxidative (excess ROS) stress in pathogenic fungi. These agents include small molecules, such as miconazole [9, 10], fluconazole [11, 12], amphotericin B (AmB) [12–16], and caspofungin [17], but also antimicrobial peptides, such as protonectin [18], baicalin [19], and various plant defensins [20–23]. To date, the induction of nitrosative (excess RNS) stress in fungal species has only been demonstrated for AmB in the pathogenic fungus *Cryptococcus gattii* [24] and for the plant defensins NaD1 and PvD1 in *C. albicans* [23, 25]. AmB belongs to the polyene class of antifungals and induces fungal cell death through apoptotic and nonapoptotic pathways [26–29]. Hence, based on the above reports, it seems that AmB can induce both excess ROS and RNS in pathogenic fungi. How the production of these different types of radicals would contribute to AmB's fungicidal action is hitherto not known. Moreover, increased insight in these AmB-induced events may lead to more efficient AmB-based therapies, as exemplified in the current study.

In this study, we further investigated the potential of AmB to induce ROS and RNS and looked at the interplay between these toxic radicals and their accumulation kinetics, thereby linking these events to AmB's killing capacity. To investigate the kinetics of the AmB-induced ROS and RNS, we used a digital microfluidic platform (DMF) in which single cells were captured and monitored over time using time lapse fluorescence microscopy. This DMF platform has been previously optimized for seeding of *Saccharomyces cerevisiae* cells and subsequently for assessing the rate by which AmB-induced membrane permeabilization events occurred at the single cell level [30]. *S. cerevisiae* has been widely used to investigate the mechanisms of action of antifungal agents, including that of AmB [14, 31–35]. Hence, also in this study, we used *S. cerevisiae* as a model organism to better understand the mode of action of AmB and translated the most prominent findings to the fungal pathogens *C. albicans* and *C. glabrata*.

## 2. Methods

### 2.1. Strains and Chemical Reagents

*Saccharomyces cerevisiae* strain BY4741, *Candida albicans* strain SC5314, and *Candida glabrata* strain BG2 were used in the cytotoxicity assays. All culture media were purchased from LabM Ltd. (Lancashire, England), unless stated otherwise. Media used were YPD (1% yeast extract; 2% peptone; and 2% glucose), 1/5 YPD (YPD diluted in distilled water), and RPMI-1640 (Roswell Park Memorial Institute-1640 medium; pH 7) with L-glutamine and without sodium bicarbonate (purchased from Sigma-Aldrich, St. Louis, MO, USA), buffered with MOPS (Sigma-Aldrich, St. Louis, MO, USA).

Amphotericin B (AmB), *N_ω_*-Nitro-L-arginine methyl ester hydrochloride (L-NAME), S-nitrosoglutathione, propidium iodide (PI), and dihydroethidium (DHE) were purchased from Sigma-Aldrich (St. Louis, MO, USA). 4-Amino-5-methylamino-2′,7′-difluorofluorescein diacetate (DAF-FM DA) was supplied by Life Technologies (Carlsbad, CA, USA). Peroxide was purchased from VWR chemicals (Radnor, PA, USA).

Fluorinert FC-40 was purchased from 3M (St. Paul, MN, USA), and chemicals for photolithopgraphy were supplied by Rohm and Haas (Marlborough, MN, USA). Fluoroalkylsilane Dynasylan® F 8263 was supplied by Evonik (Essen, Germany). AZ1505 photoresist and Teflon-AF® were obtained from Microchemicals GmbH (Ulm, Germany) and Dupont (Wilmington, DE, USA), respectively. Parylene C dimer and Silane A174 were purchased from Plasma Parylene Coating Services (Rosenheim, Germany).

### 2.2. Cell Culture Conditions

*S. cerevisiae*, *C. albicans*, or *C. glabrata*, grown overnight in YPD at 30°C and 250 rpm, were diluted to an optical density (OD) of 0.15 measured at *λ* = 600 nm in a flask containing 50 mL of fresh YPD and further cultured for 5 h at 30°C and 250 rpm (*S. cerevisiae*) or 37°C and 200 rpm (*Candida* spp.), to obtain exponentially growing cells. Cells were then pelleted by centrifugation (3 min, 4000 rpm), washed and resuspended in 1/5 YPD for *S. cerevisiae* or RPMI-1640 medium for *C. albicans* and *C. glabrata* to an OD of 3 for further use in the experiments.

### 2.3. Cytotoxicity Assays in Bulk

Exponentially growing cells were supplemented with PI, DHE, or DAF-FM DA to a final concentration of 3 *μ*M, 17 *μ*M, and 5 *μ*M, respectively, and subsequently treated either with DMSO or water (controls), a range of AmB dosages (0.625 *μ*M–10 *μ*M, dissolved in DMSO), 200 mM L-NAME (dissolved in water), or a combination of the above, with a final DMSO concentration of 1% (*v*/*v* %). After mixing, the cell suspensions were transferred to Eppendorf tubes, covered with a layer of silicon oil, placed on a horizontal shaker at 5 rpm, and incubated in the dark for 3 h at room temperature to be compliant with the DMF setup. In case of *C. albicans* and *C. glabrata*, however, the assays were performed at 37°C to be clinically relevant. A plating assay was carried out at the start of the treatment to account for the number of cells at this point. After 3 h, cells were pelleted (3 min, 4000 rpm), washed and resuspended in phosphate buffered saline (PBS), and subsequently plated or subjected to flow cytometry on a BD Influx™ cell sorter. In the plating assay, a 10-fold dilution series of the cell suspensions was prepared in PBS and appropriate cell suspensions were spread in YPD agar plates, after which the plates were allowed to dry for 10 min and incubated for 48 h at 30°C to visualize the number of colony forming units (CFUs). For flow cytometry, cells were monitored for fluorescence at 540/608 nm (FL3 610_20), 485/515 nm (FL1 580_30), or 495/515 nm (FL2 530_40) for detection of membrane permeabilization with PI, detection of superoxide radical accumulation with DHE, or detection of nitric oxide radical accumulation with DAF-FM DA, respectively.

### 2.4. Cell Cycle Analysis

Aliquots of cells were collected at the indicated time points and cells were pelleted, washed, and fixed with ethanol (70% *v*/*v*) for at least 30 min at 4°C. Cells were then resuspended in sodium citrate buffer (50 mM sodium citrate, pH 7.5), sonicated and treated with RNAse for 1 h at 50°C, followed by subsequent incubation with 20 *μ*g/ml proteinase K for 1 hour at 50°C. Cell DNA was then stained overnight with SYBR Green 10,000 x (Molecular Probes/Invitrogen, Carlsbad, CA), diluted 10-fold in Tris-EDTA (pH 8.0), and incubated overnight at 4°C. Before flow cytometry analysis, samples were diluted 1 :  4 in sodium citrate buffer. The SYBR signals were measured using a BD LSR II™ (Becton Dickinson, NJ, USA) with a 488 nm excitation laser. Signals from 30,000 cells/sample were captured in FITC channel (530 nm ± 30 nm), at a flow rate of about 1000 cells/s. The percentage of cells in each phase of the cell cycle was determined offline with ModFit LT software (Verity Software House, Topsham, ME).

### 2.5. Checkerboard Antifungal Assays

AmB (dissolved in DMSO), L-NAME (dissolved in water), and S-nitrosoglutathione (dissolved in water) were 2-fold serially diluted across the columns and rows of a 96-well microtiter plate. Subsequently, AmB dilutions were further diluted 10-fold in 1/5 YPD. Next, 20 *μ*l volumes of these dilutions were transferred to a microtiter plate, allowing the analysis of unique combinations of two compounds. Exponentially growing *S. cerevisiae* cells were diluted to an OD of 0.10, measured at *λ* = 600 nm, in 1/5 YPD, and subsequently, 80 *μ*l was added to the microtiter plate, resulting in a final DMSO concentration of 1% (*v*/*v* %). In parallel, the number of colony forming units (CFU) of this exponential culture was determined by plating assay. After 24 h of incubation at 30°C, the OD of the checkerboard plate was measured at *λ* = 600 nm, to examine the growth of *S. cerevisiae* cells. Subsequently, a plating assay was performed of specific wells of the checkerboard assay to assess cell viability of specific AmB-L-NAME combinations. Based on preliminary data of this experiment, we have performed power calculations (*α* = 0.05; *b* = 0.8) for an AmB concentration of 0.313 *μ*M, which is the tested value closest to the IC50 (0.282 *μ*M AmB for *S. cerevisiae*). These calculations indicated that we needed 2 biological replicates to assure a power of *b* = 0.8. Recalculations of the power calculations, based on data of the 2 biologically independent experiments, confirmed the previously executed power calculations.

### 2.6. Fabrication of Digital Microfluidic Plates

Digital microfluidic plates were fabricated as described previously [30]. The assembly consists of an actuation plate and a grounding plate, as presented in Supplemental Information S3 Figure available online at https://doi.org/10.1155/2017/4064628. For fabricating the actuation plate (S3 Figure A), cleaned glass wafers (1.1 mm) were sputter coated with chromium (100 nm) and patterned using standard photolithographic processes. After cleaning the plates with acetone and isopropyl alcohol twice, the surface was plasma activated (O_2_ plasma, 150 mtorr, 100 W) and the plates were primed with Silane A174 to promote adhesion of the parylene C layer (3 *μ*m) that was subsequently coated using chemical vapour deposition. Next, a thin layer of Teflon-AF (200 nm, using 3% *w*/*w* in Fluorinert FC-40) was spin-coated (1200 rpm) on top of the parylene C layer and baked for 5 min at 110°C and 5 min at 200°C. Crenelated actuation electrodes with dimensions of 2.8 mm × 2.8 mm were selectively actuated to manipulate individual droplets of 2.7 *μ*L, using customized software.

For fabrication of the grounding plate (Supplemental Information S3A Figure) of the DMF device, cleaned glass wafers (1 mm) were coated with an aluminium layer (40 nm) using thermal evaporation, leaving two 2.5 × 2.5 mm visualization windows. Fluoroalkylsilane Dynasylan F 8263 was coated on the aluminium to improve adhesion of the subsequent spin-coated Teflon-AF layer (3 *μ*m). Microwells were patterned in the Teflon-AF layer following a hard contact masking procedure, developed by depositing parylene C (1 *μ*m) and aluminium (60–80 nm) layers. A thin layer of AZ1505 photoresist was spin-coated on the aluminium layer, and the aluminium was patterned and etched using standard photolithography processes. The pattern was then transferred from the aluminum to the Teflon-AF using O_2_ plasma (150 mtorr, 100 W) for 10 min. Finally, using a dry lift-off method, the aluminium-parylene C mask was peeled off using a pair of forceps, revealing two microwell arrays (1.9 mm × 1.9 mm) on a single grounding plate, consisting of 22,000 microwells each, arranged in a hexagonal pattern with a pitch distance of 14 *μ*m [36]. The dimensions of the microwells were measured to be approximately 5.5 *μ*m in width and 3 *μ*m in depth.

### 2.7. Cytotoxicity Assays on DMF Platform

A schematic overview of the cytotoxicity assays on the DMF platform is given in Supplemental Information S3B Figure. Exponentially growing cells were supplemented with PI or DHE to a final concentration of 3 *μ*M and 17 *μ*M, respectively, and subsequently treated either with DMSO or water (controls), a range of AmB dosages (5 *μ*M and 10 *μ*M, dissolved in DMSO), 200 mM L-NAME (dissolved in water), or a combination of the above, with a final DMSO concentration of 1% (*v*/*v* %). After 5 min, two droplets, one containing the mixed cell suspension and one containing the corresponding composition without cells, were placed on two separate electrodes of the actuation plate. The actuation and grounding plate were assembled, thereby aligning the microwell array with the cell droplet and sandwiching it between the plates. To prevent sticking and evaporation of the droplets, 80 *μ*L of silicon oil was added between the plates by pipetting. The assembled plates were placed in the DMF chip holder, and the device was flipped upside down and incubated for 10 min to allow sedimentation of the cells. This step was followed by automated shuttling of the cell droplet over the microwell array for 15 times, that is, 15 seeding cycles, using software-assisted electrowetting-on-dielectric (EWOD) actuation. After seeding, the cell droplet was actuated away from the array and a droplet without cells was transferred to the array. The cell responses, that is, membrane permeabilization detected by PI or superoxide radical accumulation detected by DHE, were monitored at room temperature for 3 h in 15 min intervals using an inverted fluorescence microscope (IX-71, Olympus, Tokyo, Japan) equipped with a CCD camera. The whole array was scanned in 9 overlapping frames in approximately 15 seconds, in which a single frame covered approximately 4100 wells, using a 20x lens magnification. Both fluorescence and bright field images were collected using the same excitation/emission wavelengths as described above.

### 2.8. Calculation of Fluorescence Intensity per Cell

The DMF array was imaged for 3 h in 15 min intervals (i.e., 12 time points), and images were processed in ImageJ (v1.47, NIH, MD) for background correction using rolling ball algorithm with a radius of 50 pixels. Salt-and-pepper noise was removed using the despeckle option in ImageJ. Next, the images were loaded in MATLAB (The Mathworks, Natick, MA), and a custom MATLAB code was executed. The single image captured at 180 min was processed by MATLAB to identify the single fluorescent cells in contrast with the background. The MATLAB code detected the area of a single cell, and a unique numerical digit was allotted to each cell. Within the detected area of a single cell, the maximum pixel value was registered together with its respective coordinate in a vector array. Next, the MATLAB code was executed on all the images captured between 15 min and 165 min. The fluorescence intensity of each individual cell in different time frames was monitored by detecting the pixel values for the registered coordinates. The final output was tabular data with pixel intensities of single cells identified with unique numerical digits, as detected in 12 consecutive time points.

### 2.9. Data Analysis

Flow cytometric data were normalized to the control data, and DMF data were normalized to the first data point, that is, 15 min. For plating assays, the number of CFUs per mL was displayed in Log scale. Data were analysed with GraphPad Prism 6 SPPS (GraphPad Software, Inc., CA, USA). Two-way ANOVA followed by Dunnett multiple comparison test was performed to analyse statistically significant differences in the number of PI-, DHE-, and DAF-FM DA-positive cells and cells able to proliferate between control and different AmB treatments in *S. cerevisiae*, *C. albicans*, and *C. glabrata*. Pearson's product-moment correlation was performed to analyse the relation between the results obtained in the bulk and the DMF experiments. Survival analyses (Kaplan-Meier) using the Log-rank test were performed on DMF data to compare survival curves and analyse whether treatment significantly affects survival. Two-way ANOVA and subsequent Tukey or Dunnett multiple comparison tests were performed to analyse differences between bulk results for treatment with AmB in the presence or absence of L-NAME for each AmB concentration or to analyse differences in bulk results between the first data point and other data points within the same treatment, respectively. Two-way ANOVA followed by Tukey multiple comparison test was performed to analyse statistically significant differences between different treatments in the cell cycle analyses. Two-way ANOVA followed by Tukey multiple comparison test was performed to analyse statistical differences between the OD measurements for treatment with different concentrations of AmB in the absence or presence of 200 mM L-NAME or 2 mM S-nitrosoglutathione. In all cases, *P* < 0.05 was considered statistically significant. When multiple comparisons were performed, multiplicity-adjusted *P* values for each comparison are presented, taking into account the total number of groups in the ANOVA and the data in all groups.

## 3. Results

### 3.1. Amphotericin B Induced Nitric Oxide and Superoxide Radical Accumulation in *Saccharomyces cerevisiae*

First, we assessed whether AmB induces accumulation of superoxide and nitric oxide radicals in *S. cerevisiae*. To this end, yeast cultures were treated with AmB for 3 h at room temperature to be compliant with the DMF setup and subjected to flow cytometry to analyse the number of cells with increased superoxide and nitric oxide radical levels using DHE and DAF-FM DA dyes, respectively. The fluorescent probe DHE is typically used for detecting O_2_^−•^ due to its relative specificity for this ROS, with minimal oxidation induced by H_2_O_2_ or hypochlorous acid [37, 38]. Furthermore, in contrast to other intracellular dyes, there is little capacity for the formation of superoxide by DHE due to redox cycling [38]. However, nonspecific oxidation of DHE from other nonsuperoxide sources, such as cytochrome c [38], was not eliminated in this study. The number of cells with compromised plasma membranes was analysed using PI. As PI only enters cells with compromised plasma membrane, it was used as a marker to identify nonapoptotic cell death [39]. To quantify the fungicidal activity of AmB, the treated cultures were subjected to plating assays, thereby assessing the number of cells that was able to proliferate after AmB treatment.

**Figure 1:**
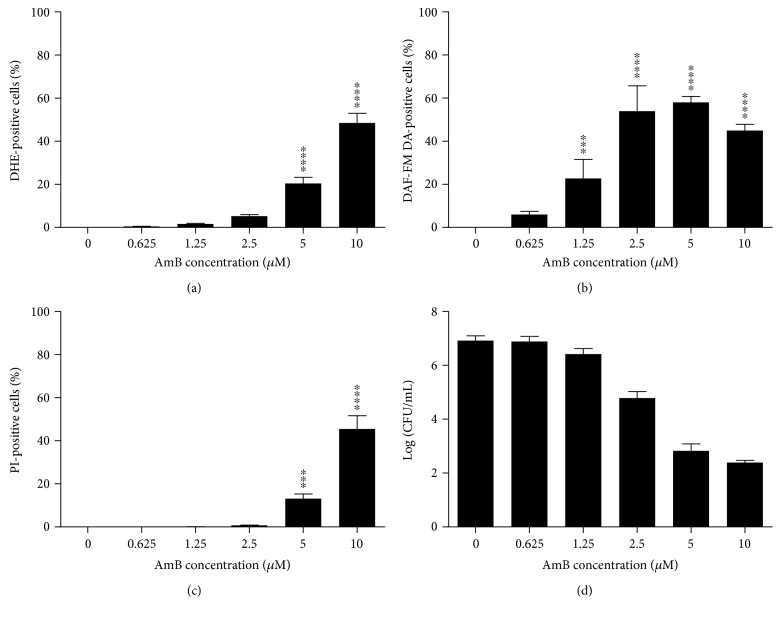
AmB induced accumulation of superoxide and nitric oxide radicals and membrane permeabilization in *S. cerevisiae*. Yeast cultures were treated with different concentrations of AmB for 3 h and subjected to flow cytometry or plating assays. (a) Levels of superoxide radical detected by dihydroethidium (DHE) fluorescence and flow cytometry. (b) Levels of nitric oxide radical detected by 4-amino-5-methylamino-2′,7′-difluorofluorescein diacetate (DAF-FM DA) fluorescence and flow cytometry. (c) Membrane permeabilization events detected by propidium iodide (PI) fluorescence and flow cytometry. (d) Number of CFU/mL in Log-scale, assessed by plating assays and CFU counting. Means and standard errors of the means (SEM) of at least 3 independent biological experiments (*n* ≥ 3) are presented. Two-way ANOVA followed by Dunnett multiple comparison test was performed to analyse statistically significant differences in the number of PI-, DHE-, and DAF-FM DA-positive cells and cells able to proliferate between control treatment and treatment with different concentrations of AmB. ∗∗∗ and ∗∗∗∗ represent *P* < 0.001 and *P* < 0.0001, respectively.

The number of cells that accumulated superoxide (Figure 1(a)) and nitric oxide radicals (Figure 1(b)), as well as the number of cells with permeabilized membranes (Figure 1(c)), was significantly increased by AmB treatment in a dose-dependent manner (*P* < 0.05), while the proliferative capacity of the cells was decreased, yet, not statistically significant (Figure 1(d)). This dose dependency was different for the tested responses: a maximum number of cells producing nitric oxide radicals in AmB-treated yeast cultures were found at AmB concentrations as low as 2.5 *μ*M (*P* < 0.0001) (Figure 1(b)), whereas the highest number of cells with increased superoxide radical accumulation and compromised membranes was observed at 10 *μ*M AmB (*P* < 0.0001) (Figures 1(a) and 1(c), resp.). Hence, it seemed that the production of nitric oxide radicals could be induced at AmB doses that did not trigger the accumulation of superoxide radicals or membrane permeabilization, while this resulted in a reduced proliferative capacity of cells.

### 3.2. Inhibition of Nitric Oxide Radical Production Resulted in Increased Superoxide Radical Accumulation and Loss of Proliferative Capacity by Amphotericin B

As it was previously shown that superoxide radicals react with nitric oxide radicals, resulting in strongly oxidizing RNS causing damage to proteins and nucleic acids [40–42], we investigated whether the AmB-induced superoxide radical levels could be increased by blocking the production of nitric oxide radicals. To this end, L-NAME was used. L-NAME inhibits nitric oxide synthases in mammalian cells and thus prevents the generation of nitric oxide radicals [43]. Although in yeast, only nitric oxide synthase-like enzymes are identified to date, L-NAME was shown to reduce the levels of nitric oxide radicals in yeast [44–46]. Reduction of the levels of nitric oxide radicals by L-NAME in *S. cerevisiae* was microscopically confirmed (data not shown).

**Figure 2:**
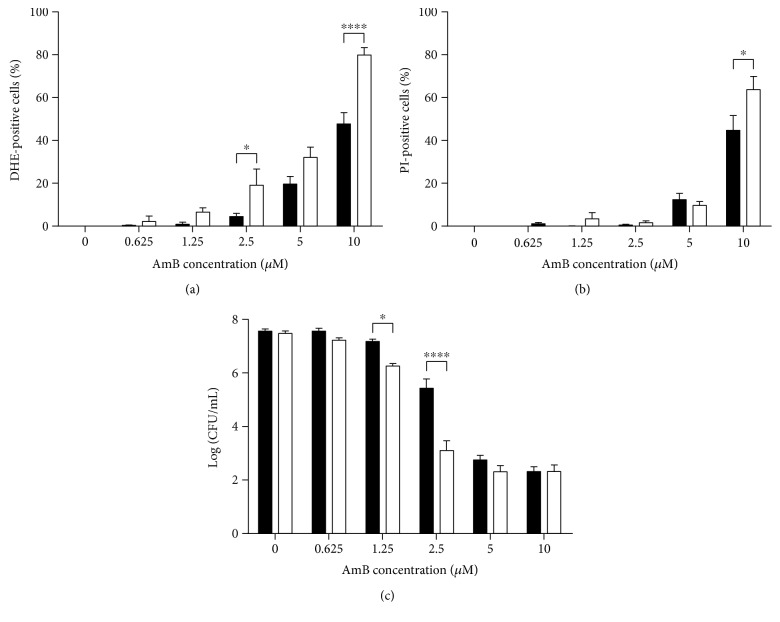
AmB-induced superoxide radical accumulation, membrane permeabilization, and loss of proliferative capacity can be increased by blocking nitric oxide radical production using L-NAME. Exponential yeast cultures were treated with different concentrations of AmB in the presence or absence of 200 mM L-NAME for 3 h. (a) Levels of superoxide radical detected by dihydroethidium (DHE) fluorescence and flow cytometry. (b) Membrane permeabilization events detected by propidium iodide (PI) fluorescence and flow cytometry. (c) Number of CFU/mL in Log-scale, assessed by plating assays and CFU counting. Means and standard errors of the means (SEMs) of at least 3 independent biological experiments (*n* ≥ 3) are presented. Black bars represent treatment with AmB alone; white bars represent treatment with AmB supplemented with 200 mM L-NAME. Two-way ANOVA followed by Tukey multiple comparison test was performed to analyse significant differences between the two treatments. ∗ and ∗∗∗∗ represent *P* < 0.05 and *P* < 0.0001, respectively. Multiplicity-adjusted *P* values are presented in the text.

In the presence of L-NAME, the number of cells with AmB-induced accumulation of superoxide radicals significantly increased as compared to that after AmB treatment alone, in the case of 2.5 *μ*M or 10 *μ*M AmB (Figure 2(a); *P* = 0.05 and *P* < 0.0001, resp.). Moreover, treatment of yeast with 1.25 *μ*M or 2.5 *μ*M AmB supplemented with L-NAME significantly reduced the number of cells that were able to proliferate, as compared to treatment with AmB alone (Figure 2(c); *P* = 0.01 and *P* < 0.0001, resp.). In contrast, only 10 *μ*M AmB with L-NAME increased the number of cells with a compromised membrane in a significant manner (*P* = 0.02), as compared to that after treatment with AmB alone (Figure 2(b)), suggesting that the combination of low concentrations of AmB with 200 mM L-NAME did not affect membrane permeabilization by AmB. In addition, inhibition of nitric oxide radical production resulted in an increased number of cells that accumulated superoxide radicals and was characterized by membrane permeabilization and inability to proliferate. These findings point towards a potential role of nitric oxide radical production in mediating tolerance towards AmB in yeast. Moreover, we have performed cytotoxicity assays with peroxide in the presence and absence of 200 mM L-NAME and found that L-NAME can only increase the killing activity of AmB but not that of peroxide, implying an AmB-specific effect of L-NAME (Supplemental Information S4 Figure).

### 3.3. Inhibition of Nitric Oxide Radical Production Resulted in Faster and Increased Superoxide Radical Accumulation and Faster Membrane Permeabilization by Amphotericin B

To gain more insights into the action of L-NAME on the kinetics of AmB-induced superoxide radical accumulation, time lapse experiments were performed on a DMF platform (for a schematic representation of the experimental setup, see Supplemental Information S3 Figure), as described in our previous study [30]. To this end, yeast was treated with either 0 *μ*M (control), 5 *μ*M, or 10 *μ*M AmB in the presence or absence of 200 mM L-NAME, as these concentrations were shown to have the most profound effect on membrane permeabilization and accumulation of superoxide radicals in the bulk experiments. During treatment, each cell was monitored over time at room temperature for 3 h in 15 min intervals for DHE or PI fluorescence. Validation of the DMF platform to monitor DHE and PI fluorescence at single cell level was performed prior to the assays described above (Supplemental Information S1 Figure).

**Figure 3:**
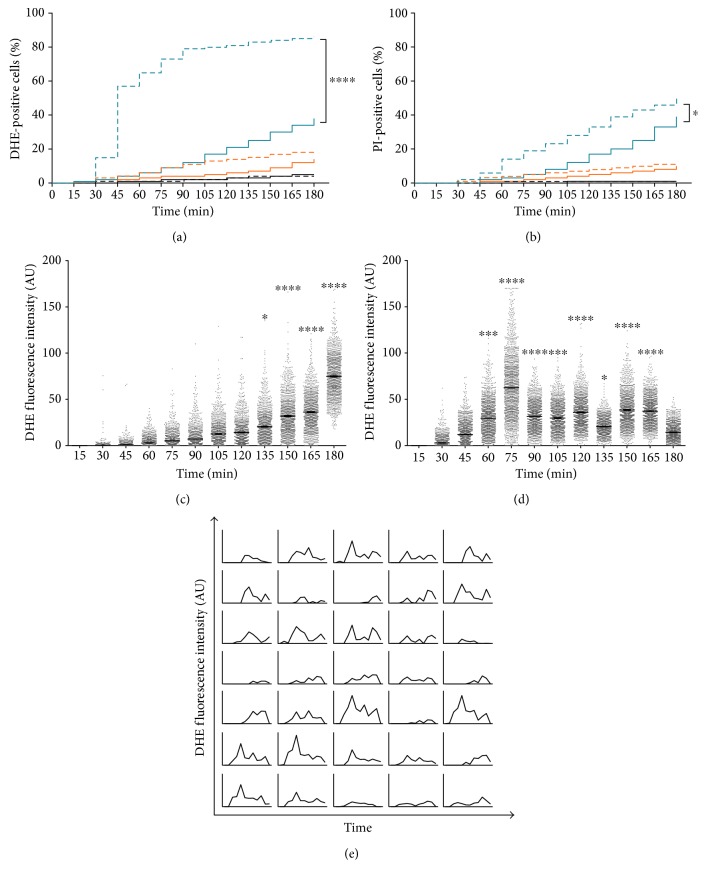
L-NAME increased and accelerated AmB-induced superoxide radical accumulation, membrane permeabilization, and intracellular superoxide radical levels. (a-b) Accumulation of superoxide radicals (a) and membrane permeabilization (b) in *S. cerevisiae* cells treated either with 0 *μ*M (black), 5 *μ*M (orange), or 10 *μ*M (blue) AmB in the presence (dashed lines) or absence (solid lines) of 200 mM L-NAME during 3 h in 15 min intervals. Log-rank tests were performed to analyse significant differences between AmB treatment and treatment of AmB in combination with 200 mM L-NAME for each AmB dose. Data of at least 3 independent biological experiments is presented (*n* ≥ 3). ∗ and ∗∗∗∗ represent *P* < 0.05 and *P* < 0.0001, respectively. (c-d) Intracellular DHE fluorescence in *S. cerevisiae* cells treated with 10 *μ*M AmB in the absence (c) or presence (d) of 200 mM L-NAME. Single cells were monitored for their DHE fluorescence during treatment for 3 h in 15 min intervals using fluorescence microscopy and the DMF platform. The fluorescence intensity of each cell is presented as arbitrary units (AU), and each dot represents a single cell. Means and standard errors of the means (SEMs) of at least 3 independent biological experiments (*n* ≥ 3), with at least 780 cells each, are presented. Two-way ANOVA followed by Tukey multiple comparison test was performed to analyse significant differences in DHE fluorescence intensity. ∗, ∗∗∗, and ∗∗∗∗ represent *P* < 0.05, *P* < 0.001, and *P* < 0.0001, respectively. (e) DHE fluorescence intensity of individual cells over time. A selection of 35 cells was randomly chosen and is representative for more than 3000 cells that were analysed in this study. Each plot represents the DHE fluorescence intensity, measured every 15 min, of one representative cell over the whole duration of the experiment, that is, 180 min.

We found an increased number of DHE- (Figure 3(a)) and PI- (Figure 3(b)) positive cells when yeast was treated with AmB supplemented with 200 mM L-NAME as compared to treatment with AmB alone, starting from 30 min to 45 min incubation, respectively. This observation was in line with the bulk results after 3 h of incubation at room temperature that were obtained by flow cytometry (Figure 2).

Survival analyses were performed to test the hypothesis that different treatments (i.e., AmB in the presence or absence of L-NAME) affect survival in a significantly different manner, in which survival is defined as the occurrence of a specific event [47]. Here, we analysed whether the occurrence of superoxide radical accumulation and membrane permeabilization upon treatment with AmB in the presence and absence of L-NAME was significantly different. The survival curves for treatment with 10 *μ*M AmB in the presence or absence of 200 mM L-NAME were significantly different in both DHE and PI experiments (*P* < 0.0001) (Figures 3(a) and 3(b)), indicating that L-NAME significantly affected the number of superoxide radical accumulating cells and the number of membrane permeabilization events induced by AmB over time. When cells were treated with 10 *μ*M AmB in combination with 200 mM L-NAME, a median survival of 45 min was observed in the DHE experiments, that is, 50% of the cells was DHE positive after 45 min of treatment. In contrast, when AmB was applied alone, 50% of DHE-positive cells in the treated yeast culture was not reached after 180 min (median survival > 180 min), which implied that AmB-induced superoxide radical accumulation occurred faster in cells treated in the presence of L-NAME. Additionally, a hazard ratio (Log-rank) of 4.21 was found when comparing the survival curve of cells treated with 10 *μ*M AmB supplemented with 200 mM L-NAME to that of cells treated with 10 *μ*M AmB, indicating that the rate by which superoxide radicals were formed is 4.21 times faster in the combination treatment, compared to treatment with AmB alone. The same was true for membrane permeabilization events induced by AmB supplemented with L-NAME; upon treatment with the latter, a median survival of 180 min was observed, as compared to >180 min for treatment with AmB alone, and when comparing both survival curves, a hazard ratio of 1.56 was found. This suggested also that membrane permeabilization occurred faster when cells were subjected to AmB in the presence of L-NAME, as compared to treatment with AmB alone.

We further confirmed that the fast increase in superoxide radical levels during AmB-L-NAME combination treatment was linked to a block in the production of nitric oxide radicals. As we were unable to monitor nitric oxide radicals over time using the DMF platform due to an incompatibility of the DAF-FM DA dye and the DMF setup, we opted to further investigate the kinetics of nitric oxide radical production by flow cytometry. Indeed, upon AmB treatment, yeast cells started to produce nitric oxide radicals from 30 min onwards (*P* < 0.0001), and a similar trend was observed to that of superoxide radical accumulation during treatment with AmB supplemented with L-NAME (Supplemental Information S2 Figure).

To further elucidate the variation of superoxide radical levels when cells were subjected to AmB treatment in the presence of L-NAME, we analysed the fluorescence intensity of individual cells. To this end, single cells were monitored over time in 15 min intervals, and hence the fluorescence intensity of each cell, represented by one dot, was reanalysed every 15 min. We found that the DHE fluorescence intensity of cells during AmB treatment gradually increased over time, and the highest fluorescence intensity was measured at 180 min, the end point of this study (Figure 3(c)). Compared to the DHE fluorescence at 15 min, the DHE fluorescence intensity was significantly different from 135 min onwards. In contrast, the DHE fluorescence intensity of cells treated with 10 *μ*M AmB supplemented with 200 mM L-NAME showed two subpopulations, suggesting that superoxide radical accumulation took place in a biphasic manner; the first and highest superoxide radical accumulation peak was observed at approximately 75 min, followed by a rather slow decrease and a second peak at approximately 150 min (Figure 3(d)). Here, the DHE fluorescence intensity was statistically significant from 60 min onwards (compared to DHE fluorescence at 15 min). The kinetics of DHE fluorescence of 35 individual cells, representative for more than 3000 analysed cells, showed different subsets of cells in ROS readouts over time. Some subsets showed an increase in DHE fluorescence, followed by a decrease in fluorescence at certain time points, while other subsets showed a gradual increase in fluorescence over time (Figure 3(e)). Hence, it seemed that not only the number of cells accumulating superoxide radicals increased when subjected to AmB treatment in the presence of L-NAME, but also the intracellular levels of superoxide radicals were altered in a time-dependent manner, as compared to treatment of cells with AmB alone.

Moreover, to further support the data of the single cell analysis via the DMF platform, we performed additional time lapse experiments in bulk via FACS and analysed the subpopulations of DHE- and PI-positive cells of cultures treated with 10 *μ*M AmB in the presence and absence of 200 mM L-NAME for 30, 60, 90, and 180 min. We found an increased number of DHE- and PI-positive cells when yeast was treated with 10 *μ*M AmB supplemented with 200 mM L-NAME as compared to treatment with AmB alone, starting from 30 min to 90 min incubation, respectively (Supplemental Information S5 Figure). These bulk data showed faster and increased ROS accumulation and faster membrane permeabilization by AmB when coincubated with L-NAME and corroborated the data of the single cell analysis via the DMF platform. Note that the number of PI-positive cells induced by AmB in the presence of L-NAME is higher when assessed in bulk as compared to the DMF setup.

### 3.4. Inhibition of Nitric Oxide Radical Accumulation Resulted in Faster Arrest of Proliferative Capacity of *Saccharomyces cerevisiae* Cells by Amphotericin B

The results described above indicate that AmB-induced superoxide radical accumulation and membrane permeabilization were significantly altered upon the addition of L-NAME. This tempted us to further investigate whether the proliferative capacity of cells was affected in a time-dependent manner, when comparing both treatments. To this end, plating of *S. cerevisiae* cultures subjected to both treatments was carried out every 15 min, and the number of cells that were able to proliferate (and form CFU) was determined.

**Figure 4:**
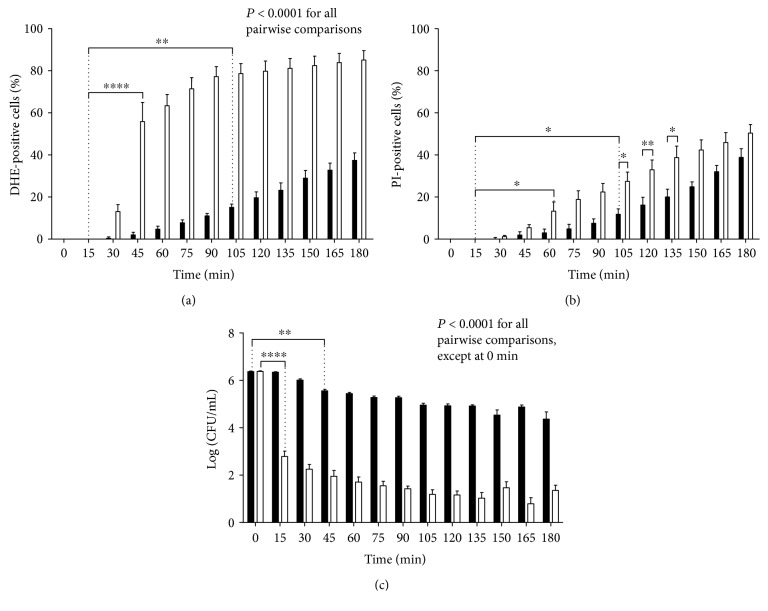
L-NAME decreased the proliferative capacity of cells during AmB treatment, which seems independent of superoxide radical accumulation. Exponential yeast cells were treated with 10 *μ*M AmB in the presence (white bars) or absence (black bars) of 200 mM L-NAME for 3 h. Cells were analysed for their DHE and PI fluorescence in the DMF setup (a and b) or subjected to bulk plating assays (c) every 15 min. Means and standard error of the means (SEMs) of at least 3 independent biological experiments (*n* ≥ 3) are presented. Two-way ANOVA followed by Tukey multiple comparison test was performed to analyse significant differences between the two treatments; Two-way ANOVA followed by Dunnett multiple comparison test was performed to analyse significant differences between the first data point (i.e., 0 min (in (c)) or 15 min (in (a) and (b))) and other data points within the same treatment (only the primary significant difference is presented to avoid overcrowding of the figure); ∗, ∗∗, and ∗∗∗∗ represent *P* < 0.05, *P* < 0.01, and *P* < 0.0001, respectively. A dotted line is shown at 15 min to point out the clear differences between the responses at this time point.

At all time points, the proliferative capacity of cells treated with 10 *μ*M AmB and 200 mM L-NAME was significantly reduced as compared to cells treated with 10 *μ*M AmB alone (*P* < 0.0001) (Figure 4(c)). In addition, it seemed that the proliferative capacity of cells subjected to AmB-L-NAME treatment was reduced very fast, that is, within 15 min (*P* < 0.0001), whereas the proliferative capacity of cells upon treatment with AmB alone decreased in a significant manner from 45 min onwards (*P* = 0.003). This suggested that the fast decrease in proliferative capacity of cells within 15 min upon incubation with AmB and L-NAME was independent of superoxide radical accumulation and membrane permeabilization. Similar observations were made in the survival analyses for superoxide radical accumulation and membrane compromising events (Figures 3(a) and 3(b)) and were supported by a second statistical analysis (Two-way ANOVA followed by Dunnett multiple comparison test). Specifically, a significant difference in the number of cells accumulating superoxide radicals (Figure 4(a)) and showing membrane permeabilization (Figure 4(b)) was found at earlier time points (i.e., 45 min versus 105 min for superoxide radical accumulation and 60 min versus 105 min for membrane permeabilization) when cells were treated with AmB in the presence of L-NAME, as compared to cells treated with AmB alone.

However, although approximately 99.5% of the treated population was not able to proliferate from 15 min onwards (Figure 4(c)) when subjected to AmB-L-NAME treatment, they were still able to accumulate superoxide radicals at that point, which resulted in a superoxide radical boost starting at 30 min (Figure 4(a)). Hence, it seemed that these cells were still metabolically active and possibly used increased intracellular superoxide radical levels to enter a programmed cell death pathway. In contrast, loss of proliferative capacity of cells treated with AmB alone (Figure 4(c)) might be explained by the gradual increase in the number of membrane permeabilization events; a similar trend in both curves was observed (Figure 4(b)).

**Figure 5:**
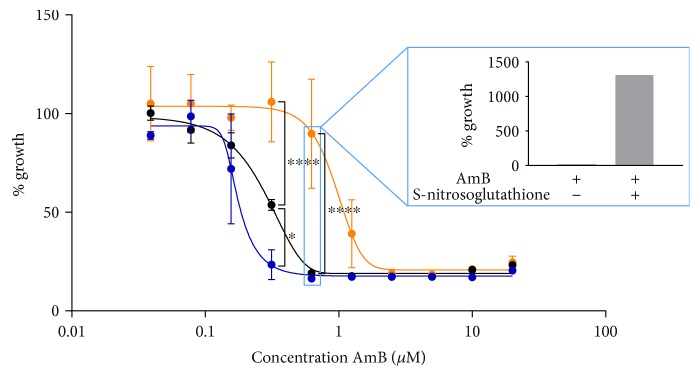
The nitric oxide donor, S-nitrosoglutathione, inhibited the killing activity of AmB. Yeast cells were treated with different concentrations of AmB, in the absence (black) or presence of 200 mM L-NAME (blue) or 2 mM S-nitrosoglutathione (orange). Means and standard errors of the means (SEMs) of at least two independent biological experiments (*n* ≥ 2) are presented. The number of CFU/mL for different treatments (insert) was assessed by plating assays and CFU counting and is shown relative to the number of CFU/mL at the start of the experiment. Two-way ANOVA followed by Tukey multiple comparison test was performed to analyse significant differences between the two treatments; ∗ and ∗∗∗∗ represent *P* < 0.05 and *P* < 0.0001, respectively.

To further confirm the crucial role of nitric oxide in modulating AmB's fungicidal activity, we performed checkerboard assays with AmB and the nitric oxide donor, S-nitrosoglutathione. We found that 2 mM S-nitrosoglutathione reduced the activity of AmB (Figure 5). Hence, nitric oxide plays an important role in modulating AmB's activity.

### 3.5. Amphotericin B Induced Cell Cycle Arrest in *Saccharomyces cerevisiae* Independently of L-NAME

We further analysed whether the increased loss of proliferative capacity upon combined AmB and L-NAME treatment within 15 min of treatment can be attributed to cell cycle arrest. To this end, we analysed the fraction of cells in the G0/G1, S, and G2/M cell cycle phases at the beginning and after 7.5 and 15 min of incubation with either AmB alone or AmB supplemented with L-NAME.

**Figure 6:**
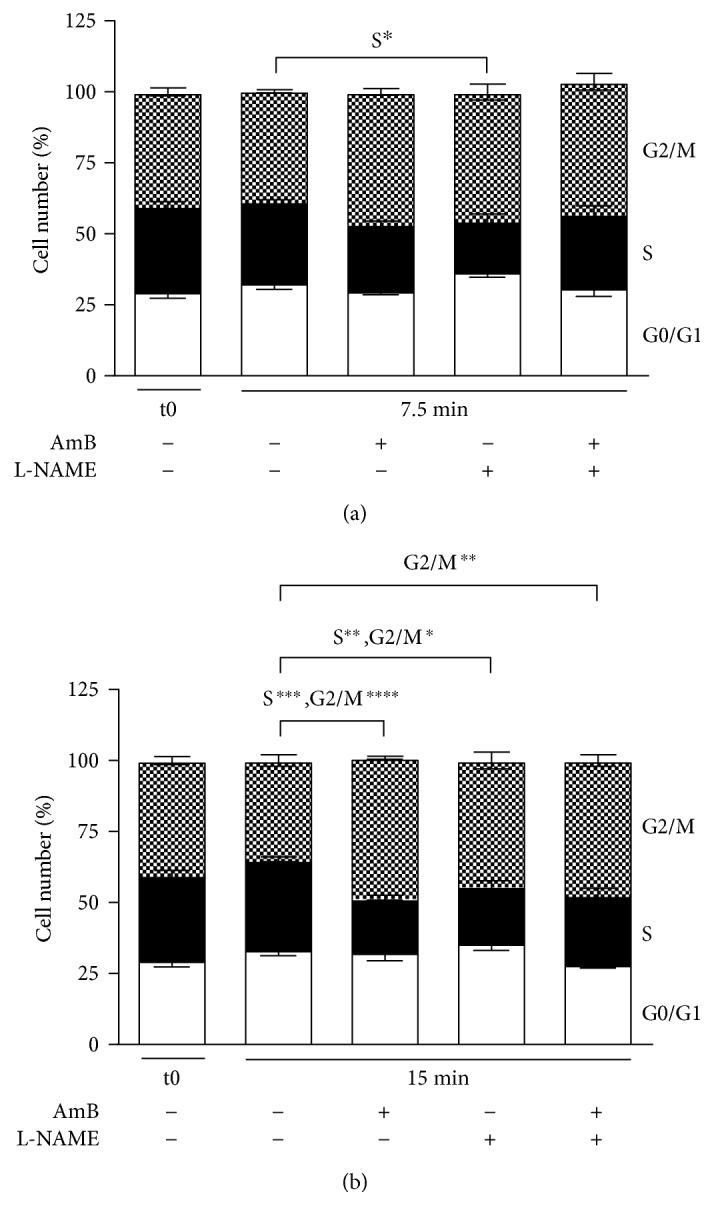
Amphotericin B induced cell cycle arrest in the G2/M phase in yeast. Exponential yeast cultures were treated with either control (1% DMSO; 10% mQ), 200 mM L-NAME (dissolved in mQ), 10 *μ*M AmB (dissolved in DMSO), or a combination of the above for 7.5 min (a) and 15 min (b). After treatment, cells were washed with PBS, fixed in 70% EtOH, stained with PI, and subjected to flow cytometry for cell cycle analysis. White bars represent cells in the G0/G1 phase, black bars represent cells in the S phase, and pixelated bars represent cells in the G2/M phase. Means and standard error of the means (SEMs) of 3 independent biological experiments (*n* = 3) are presented. Two-way ANOVA followed by Tukey multiple comparison test was performed to analyse differences between the cell cycle distributions of control treatment and AmB, L-NAME, or AmB + L-NAME treatment and between cell cycle distributions of AmB treatment and AmB + L-NAME treatment. ∗, ∗∗, ∗∗∗, and ∗∗∗∗ represent *P* < 0.05, *P* < 0.01, *P* < 0.001, and *P* < 0.0001, respectively. Multiplicity-adjusted *P* values are presented in the main text.

AmB induced cell cycle arrest in the G2/M phase in yeast at 15 min of treatment, as compared to control cells (*P* < 0.0001) (Figure 6). Interestingly, treatment with L-NAME alone resulted in a decrease in the amount of cells in the S phase at 7.5 and 15 min (*P* = 0.03 and *P* = 0.0009, resp.) and an increase in the amount of cells in the G2/M phase at 15 min (*P* = 0.03), compared to control-treated cells. Yet, L-NAME alone did not increase the other cell cycle phase distributions in a significant manner at both time points, compared to control-treated cells (*P* > 0.05 at 7.5 min and 15 min of treatment). Treatment with AmB-L-NAME did not significantly alter cell cycle phase distributions as compared to those in treatment with AmB alone, suggesting that the observed increased loss of proliferative capacity of yeast cells treated with the AmB-L-NAME combination, compared to AmB treatment alone, was not due to increased cell cycle arrest.

### 3.6. *Candida albicans* and *Candida glabrata* Were More Susceptible to Amphotericin B Treatment in the Presence of Nitric Oxide Radical Production Inhibitors

**Figure 7:**
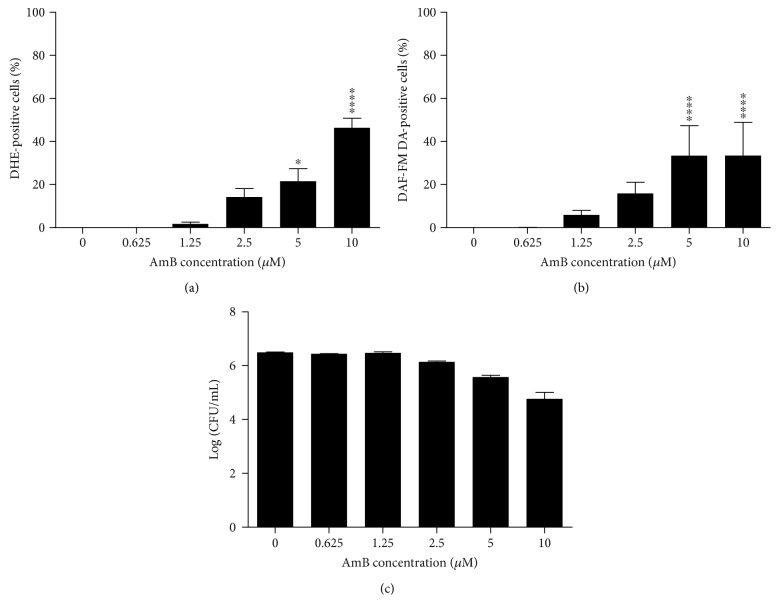
AmB induced accumulation of superoxide and nitric oxide radicals in *C. albicans* and decreased the number of cells that are able to proliferate. Exponential *C. albicans* cultures were treated with different concentrations of AmB for 3 h at room temperature and subjected to flow cytometry or plating assays. (a) Levels of superoxide radical detected by dihydroethidium (DHE) fluorescence and flow cytometry. (b) Levels of nitric oxide radical detected by 4-amino-5-methylamino-2′,7′-difluorofluorescein diacetate (DAF-FM DA) fluorescence and flow cytometry. (c) Number of CFU/mL in Log-scale, assessed by plating assays and CFU counting. Means and standard errors of the means (SEMs) of at least 3 independent biological experiments (*n* ≥ 3) are presented. Two-way ANOVA followed by Dunnett multiple comparison test was performed to analyse statistically significant differences in the number of DHE- and DAF-FM DA-positive cells and cells able to proliferate between control treatment and treatment with different concentrations of AmB. ∗ and ∗∗∗∗ represent *P* < 0.05 and *P* < 0.0001, respectively.

**Figure 8:**
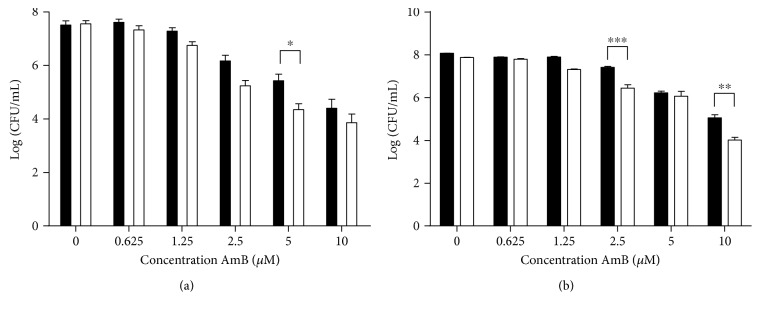
L-NAME significantly decreased the number of AmB-treated cells that are able to proliferate in *C. albicans* (a) and *C. glabrata* (b). Exponential *C. albicans* and *C. glabrata* cultures were treated with different dosages of AmB in the presence (white bars) or absence (black bars) of 200 mM L-NAME for 180 min at 37°C and subjected to plating assays. Means and standard errors of the means (SEMs) of 3 independent biological experiments (*n* = 3) are presented. Two-way ANOVA followed by Tukey multiple comparison test was performed to analyse significant differences between the two treatments; ∗, ∗∗, and ∗∗∗ represent *P* < 0.05, *P* < 0.01, and *P* < 0.001, respectively.

To validate the results obtained in yeast and in support of the clinical relevance of AmB treatment in the presence of L-NAME, we investigated the effects of this treatment on the human pathogen *Candida albicans*. We confirmed that AmB induced superoxide and nitric oxide radical accumulation (*P* < 0.0001 at 10 *μ*M AmB), associated with loss of proliferative capacity, in *C. albicans*, in a similar dose-dependent way as was observed for *S. cerevisiae* (Figure 7). These results indicated that the range of AmB concentrations used for *S. cerevisiae* was applicable for *C. albicans* as well. Furthermore, we assessed whether treatment at 37°C with AmB in the presence of L-NAME also significantly affected the number of cells that are able to proliferate as compared to treatment with AmB alone. At 37°C, we found that 5 *μ*M AmB resulted in killing of the *C. albicans* culture by 2 Log units (99.00%) and 10 *μ*M AmB by 4 Log units (99.99%). These values are in line with the reported minimal fungicidal concentration (MFC) of AmB (8.66 *μ*M) in a similar experimental setup [48]. Coincubation of 200 mM L-NAME and 5 *μ*M AmB significantly reduced the number of CFUs as compared to treatment with AmB alone (*P* < 0.05) (Figure 8(a)), indicating that L-NAME also enhanced AmB's fungicidal activity against *C. albicans*. Also, in case of *C. glabrata*, we found that 10 *μ*M AmB resulted in killing of *C. glabrata* by 4 Log units (99.99%). These values are in line with the reported MFC of AmB against *C. glabrata* (17 *μ*M) [49]. Also here, 200 mM L-NAME significantly increased AmB's fungicidal activity against *C. glabrata* (Figure 8(b)). All these data point to the clinical potential of combining AmB with L-NAME.

## 4. Discussion

**Figure 9:**
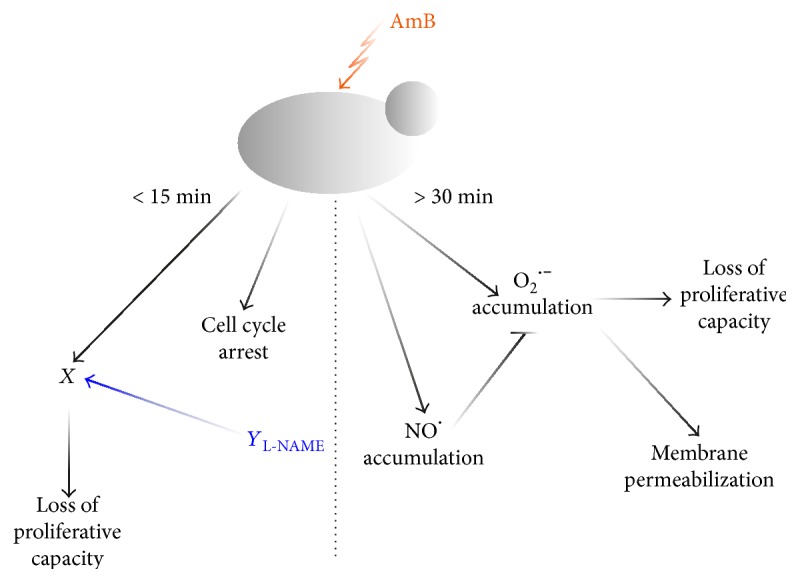
Schematic overview of the major findings on AmB mechanism of action in this study. Within 15 min, AmB caused cell cycle arrest in the G2/M phase and induced a yet to be elucidated event *X*, the latter leading to loss of proliferative capacity in yeast. These effects were independent of nitric oxide radicals, superoxide anion radicals, and membrane permeabilization. After 30 min, AmB induced the accumulation of superoxide radicals, which was associated with membrane permeabilization and loss of proliferative capacity in yeast, and was partially blocked by beneficial action of nitric oxide radicals. Interestingly, the combinatorial action of AmB and L-NAME induced a yet to be identified event *Y* within 15 min, which was independent of nitric oxide radicals, and enhanced the effect of event *X*, leading to enhanced loss of proliferative capacity in yeast.

The aim of this study was to investigate and understand how AmB-induced oxidative and nitrosative stresses (characterized by excess of superoxide radicals and nitric oxide radicals, resp.) are linked to fungal cell death. To inhibit the generation of nitric oxide radicals and nitrosative stress in cells, we used the nitric oxide synthase inhibitor L-NAME. From the bulk studies, we found that superoxide radical accumulation increased when nitric oxide production was inhibited, thereby increasing AmB's antifungal activity. We then further assessed the kinetics of superoxide radical accumulation, membrane permeabilization, and loss of proliferative capacity using a DMF platform in which individual *S. cerevisiae* cells were captured and monitored for their responses over time during treatment. As seeding of *C. albicans* was problematic due to the presence of hyphae, we first tested our hypotheses on *S. cerevisiae* and translated the most prominent findings to *C. albicans* and *C. glabrata* afterwards using bulk assays. We showed that L-NAME increased and accelerated the effect of AmB on the accumulation of superoxide radicals, membrane permeabilization, and loss of proliferative capacity in *S. cerevisiae*. Moreover, we showed that the data obtained via time lapse experiments in bulk corroborates the data of the single-cell analysis via the DMF platform (Supplemental Information S5 Figure). We revealed that superoxide radicals are important mediators for AmB-induced fungal cell death. However, L-NAME could only increase the killing potential of AmB, but not that of peroxide. This implies an AmB-specific effect of L-NAME and might point to L-NAME's effects via an ergosterol-dependent pathway. Indeed, ROS generation by AmB has been described as a consequence of AmB's spontaneous insertion into ergosterol-containing membranes [50, 51]. In contrast, nitric oxide radicals seemed to play a role in mediating tolerance towards AmB, pointing to a beneficial role of nitric oxide radicals in the yeast response towards AmB. We found that cellular responses are classified into two groups based on the time point that they occur, that is, within 15 min and from 30 to 45 min onwards (Figure 9).

Upon treatment of *S. cerevisiae* with AmB in the presence of L-NAME, not only an increased level of superoxide radicals was found as compared to treatment with AmB alone, but also an accelerating effect on these levels was observed (Figures 3(a) and 3(b)). Our DMF approach, allowing a detailed kinetic study at a single-cell level, showed that superoxide radicals accumulated in a biphasic manner during AmB treatment in the presence of L-NAME, resulting in two superoxide radical accumulation peaks at 75 min and 150 min, respectively. This was not observed for cells treated with AmB in the absence of L-NAME, where a superoxide radical accumulation peak seemed to manifest at 180 min, the endpoint of this study (Figures 3(c) and 3(d)). Interestingly, all cell responses, being superoxide radical accumulation, membrane permeabilization, and loss of proliferative capacity, presented themselves significantly faster, as compared to these responses during treatment with AmB alone. Therefore, it might well be that L-NAME solely accelerated AmB action with respect to superoxide radical accumulation and membrane permeabilization, and hence, similar outcomes might be expected for treatment with AmB alone over a longer period of time (i.e., >180 min). Whether, this is the case that requires further investigation. This hypothesis is supported by the fact that L-NAME acts fungistatic (MIC against *S. cerevisiae* of 250 mM), however not fungicidal when administered alone (Supplemental Information S6 Figure), and does not affect the level of superoxide radicals, membrane permeabilization, and proliferative capacity of control cells (Figure 2), suggesting that the observed effect on cell responses is not caused by a similar and dual action of L-NAME and AmB, as is often the case for synergistic interactions.

Secondly, and most notably, L-NAME had a strong enhancing effect on AmB-induced loss of proliferative capacity in yeast: within 15 min, approximately 99.5% of the cells lost their proliferative capacity when subjected to AmB treatment in the presence of L-NAME. In contrast, treatment with AmB alone did not reach a similar negative impact on proliferative capacity of cells within 180 min (Figure 4(c)). This suggest also that here nitric oxide radicals play an important, beneficial, role in the response towards AmB. Yet, we showed that nitric oxide radicals only accumulated from 30 min onwards (Supplemental Information S2 Figure). Hence, L-NAME seemed to have an additional effect apart from inhibiting nitric oxide radical production, resulting in enhancement of AmB fungicidal activity, and this effect occurred within 15 min of treatment (event *Y* in Figure 9). Interestingly, cells receiving treatment with AmB and L-NAME were able to accumulate superoxide radicals only after 30 min, suggesting that these accumulated positive cells were still metabolically active and that AmB-L-NAME-treated cells might use increased levels of superoxide radicals, and thus oxidative stress, to enter a programmed cell death pathway. In addition, as the rapid loss of proliferative capacity of cells upon combined treatment with AmB and L-NAME could not be explained by superoxide radical accumulation and membrane permeabilization, it seems that mechanisms other than these underlie the negative effect on the proliferative capacity of cells during the first 15 min of AmB treatment in the presence of L-NAME (i.e., event X in Figure 9). A plausible explanation for the loss of proliferative capacity, independent of oxidative stress and nonapoptotic cell death, is cell cycle arrest. We showed that AmB induced cell cycle arrest in the G2/M phase in yeast within 15 min of treatment. These data are in line with other reports on the effect of AmB on the cell cycle in mammalian cell lines [52, 53]. However, this effect was found to be independent of L-NAME, indicating that cell cycle arrest could not account for the observed increased loss of proliferative capacity when cells were treated with the AmB-L-NAME combination.

Overall, it seems that nitric oxide radicals play a beneficial role in AmB antifungal activity, as further demonstrated by the S-nitrosoglutathione-induced inhibition of AmB's killing activity (Figure 5). Nitric oxide radicals were previously shown to protect bacteria against a wide spectrum of antibiotics by alleviating the oxidative stress imposed by them [54]. In addition, nitric oxide radicals were reported to affect fungal cell death, both in beneficial and destructive manners. Specifically, increased intracellular nitric oxide radical levels are suggested to play a cytoprotective role in yeast during stress from heat-shock and hydrostatic pressure [55]. In contrast, PAF26-induced production of nitric oxide radicals was correlated to its antifungal activity, and administering L-NAME partially restored yeast growth in the presence of PAF26, indicating that nitric oxide radicals play an important role in PAF26-induced cell death [44]. In line, Almeida and colleagues showed that nitric oxide is a crucial mediator of H_2_O_2_-induced apoptosis in yeast and that blockage of nitric oxide radical production by L-NAME decreased the intracellular levels of ROS, thereby increasing survival [46]. Interestingly, in our study, L-NAME increased the accumulation of superoxide radicals during AmB treatment, while decreasing the proliferative capacity of cells in the presence of AmB, and thus decreasing survival. It seems that a nitric oxide radical-dependent tolerance system is switched on upon AmB treatment in yeast, perhaps similar to the system recently described by Nasuno and colleagues [56]. In that study, a downstream pathway of nitric oxide radicals involved in high-temperature stress tolerance in yeast was unravelled. They showed that nitric oxide radicals activated the transcription factor Mac1 that on its turn induced the *CTR1* gene and resulted in increased cellular copper levels, which then resulted in activation of Sod1, a superoxide dismutase [56]. Alternatively, it could also be that nitric oxide activates, potentially via S-nitrosylation, AmB tolerance pathways such as the yeast HOG pathway [57, 58]. How exactly tolerance to AmB via nitric oxide production is mediated requires further investigation.

We further translated the most prominent findings to the human pathogens, *Candida albicans* and *Candida glabrata*, and found that treatment of *C. albicans* or *C. glabrata* with AmB in the presence of L-NAME significantly increases the loss of proliferative capacity, as compared to treatment with AmB alone, suggesting that treatment of AmB in the presence of L-NAME might have a clinical relevance. L-NAME has been extensively studied in *in vitro*, ex vivo, and *in vivo* systems (reviewed in [59]). It was shown to inhibit corneal angiogenesis under chemical growth factor stimulation in rabbits [60] and improve leucocyte adherence and emigration to venular endothelium, characteristic of acute inflammation, in cat jejuni [61]. In addition, L-NAME was found to modulate hemodynamics in dogs [62], ewes [63], and guinea pigs [64] and was shown to reverse sepsis-associated hypotension in various animal models [65]. In humans, L-NAME was tested to treat hypotension, asthma, and sepsis. In view of the latter, L-NAME increased the systemic vascular resistance and blood pressure in septic patients [66, 67]. In treatment of asthma, no adverse effects were found in healthy volunteers and patients with asthma, and results on exhaled nitric oxide levels indicated that L-NAME might be used for treatment of asthma [68]. Finally, L-NAME increased the mean arterial pressure and cerebral blood flow, treating hypotension in patients with tetraplegia. No adverse effects on healthy volunteers or patients were found [69–71]. Hence, although L-NAME as such is not used in a clinical setting to date, it has been studied extensively in humans during the past decades.

AmB, on the other hand, is used in clinical settings to treat invasive fungal infections. However, its applicability is limited due to its nephrotoxicity and hence, it must be used with care [26]. Recent findings indicated that AmB exerts its antifungal action by extracting ergosterol from the plasma membrane, resulting in loss of cell membrane integrity, interference with ergosterol-depending cellular processes, and ultimately cell death [29]. In addition, AmB treatment causes a significant loss of fungal replication competency and numerous morphological and physiological effects on susceptible yeast cells, including cytoplasm shrinking, abnormal nuclear and mitochondrial morphologies, and oxidative stress [72]. Finally, Teixeira-Santos and colleagues showed that pathogenic and nonpathogenic yeast cells develop compensatory responses towards AmB treatment, related to membrane polarization, metabolic activity, and ROS production, depending on the drug concentration and the duration of the treatment [14]. Likewise, we found that treatment of yeast cells with clinically relevant AmB concentrations (i.e., 0.1 *μ*M to 21.6 *μ*M AmB [14, 73]) induces the accumulation of superoxide radicals, in addition to nitric oxide radicals, in *S. cerevisiae* and *C. albicans*. Furthermore, we found that clinically relevant AmB concentrations significantly increase the loss of proliferative capacity of *S. cerevisiae*, *C. albicans*, and *C. glabrata* in the presence of L-NAME.

## 5. Conclusions

In conclusion, we showed that L-NAME can increase and accelerate AmB-induced superoxide radical accumulation and loss of proliferative capacity in *S. cerevisiae*, the latter was confirmed in the human pathogens *C. albicans* and *C. glabrata*. Moreover, we found that the production of nitric oxide radicals seems to constitute a tolerance mechanism that is induced by AmB treatment and partially counteracts AmB activity. Moreover, the combinatorial action of AmB and L-NAME induced an additional, yet to be elucidated, event that further enhanced AmB's fungicidal activity. The effects of both AmB and L-NAME have been extensively studied in various *in vitro* and *in vivo* models, pointing towards the potential of AmB-L-NAME combination treatment. However, further research on pharmacology and toxicology of the AmB-L-NAME combination needs to be performed in order to assess its potential clinical relevance.

## Supplementary Material

S1 Fig. Pearson Product-Moment Correlation analysis suggests a good linear correlation between results obtained in bulk and on the DMF platform. S2 Fig. Kinetics of nitric oxide radical production in yeast cells subjected to AmB. S3 Fig. Schematic overview of the DMF setup. S4 Fig. L-NAME does not significantly alter the killing potential of peroxide in S. cerevisiae. S5 Fig. Time lapse experiments via bulk analysis in S. cerevisiae corroborate the results of the single cell analysis via the DMF platform. S6 Fig. L-NAME acts fungistatic on S. cerevisiae.
