# Supplementary material for: Increasing the Fungicidal Action of Amphotericin B by Inhibiting the Nitric Oxide-Dependent Tolerance Pathway
Source: Oxid Med Cell Longev. 2017 Oct 10;2017:4064628. doi: 10.1155/2017/4064628 (PMC5654257; doi:10.1155/2017/4064628)
Supplement: Supplementary file 1 — S1 Fig. Pearson Product-Moment Correlation analysis suggests a good linear correlation between results obtained in bulk and on the DMF platform. S2 Fig. Kinetics of nitric oxide radical production in yeast cells subjected to AmB. S3 Fig. Schematic overview of the DMF setup. S4 Fig. L-NAME does not significantly alter the killing potential of peroxide in S. cerevisiae. S5 Fig. Time lapse experiments via bulk analysis in S. cerevisiae corroborate the results of the single cell analysis via the DMF platform. S6 Fig. L-NAME acts fungistatic on S. cerevisiae. [file 4064628.f1.docx]

**Supplemental Information**

## Increasing the fungicidal action of amphotericin B by inhibiting the nitric oxide-dependent tolerance pathway

Kim Vriens^1,#^, Phalguni Tewari Kumar^2,#^, Caroline Struyfs^1,6,#^, Pieter Spincemaille^3^, Tanne L. Cools^1^, Tadej Kokalj^2^, Belém Sampaio-Marques^4.5^, Paula Ludovico^4.5^, Jeroen Lammertyn^2^, Bruno P.A. Cammue^1,6,*^ and Karin Thevissen^1^

^1^Centre of Microbial and Plant Genetics, KU Leuven, 3001 Heverlee, Belgium

^2^BIOSYST-MEBIOS, KU Leuven, 3001 Heverlee, Belgium

^3^Department of Laboratory Medicine, University Hospitals Leuven, Herestraat 49, 3000 Leuven, Belgium

^4^Life and Health Sciences Research Institute (ICVS), School of Health Sciences, University of Minho, Braga, Portugal

^5^ICVS/3B's-PT Government Associate Laboratory, Braga/Guimarães, Portugal

^6^VIB Department of Plant Systems Biology, 9052 Ghent, Belgium

^*^To whom correspondence should be addressed: Bruno P.A. Cammue, Centre of Microbial and Plant Genetics, KU Leuven, Kasteelpark Arenberg 20, 3001 Heverlee, Belgium, Tel.: +32 16 329682; Fax.: +32 16 321966; E-mail: bruno.cammue@biw.kuleuven.be

^#^These authors contributed equally to this work

**Validation of DMF platform to monitor DHE and PI fluorescence at single cell level**

The DMF platform was validated using correlation analyses to test whether similar results were obtained on the DMF platform as for the bulk analyses by flow cytometry after 180 minutes (Fig S3). In all experiments, the Pearson correlation coefficient ranged from 0.97 to 1.00, suggesting a good correlation between bulk and DMF results in all experiments.


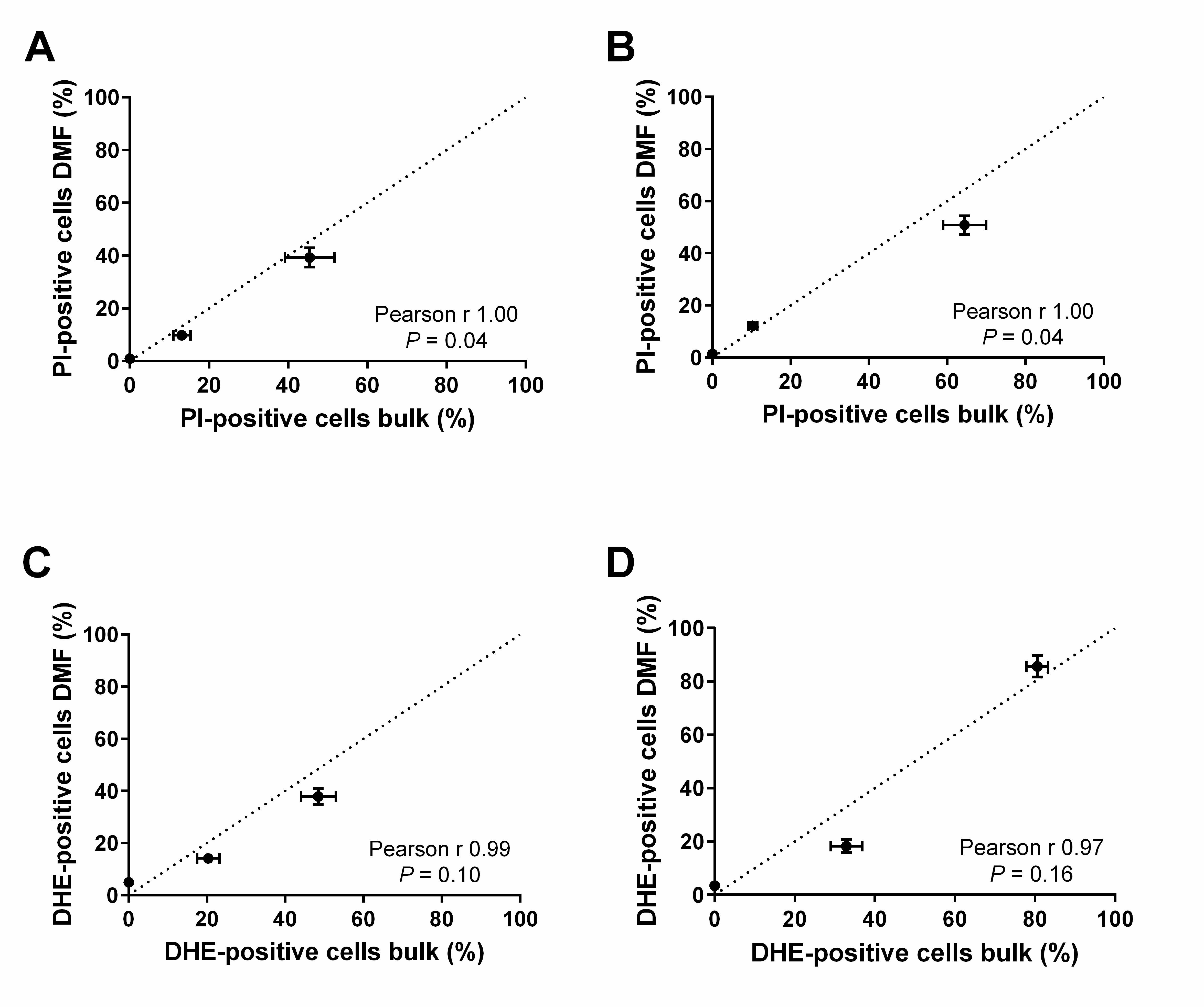


**S1 Fig. Pearson Product-Moment Correlation analysis suggests a good linear correlation between results obtained in bulk and on the DMF platform.** Exponential yeast cells were treated with 0 µM, 5 µM or 10 µM AmB in the absence (**A** and **C**) or presence (**B** and **D**) of 200 mM L-NAME for 3 hours either in bulk or on the DMF platform, after which the number of PI- or DHE-positive cells in both setups was determined and correlation analyses were performed. Means and standard errors of the means (SEMs) of at least 3 independent biological experiments are plotted (n ≥ 3). The bisector (dashed line) is a guide to the eye and does not represent a linear fit of the data.

**Kinetics of nitric oxide radical production in yeast cells subjected to AmB treatment**





**S2 Fig. Kinetics of nitric oxide radical production in yeast cells subjected to AmB.** Exponential yeast cultures were treated with either control (1% DMSO; 10% mQ; black bars) or 10 µM AmB (dissolved in DMSO; white bars) for 3 hours in bulk. Every 15 min, cells were subjected to flow cytometry to monitor their DAF-FM DA fluorescence. Means and standard error of the means (SEM) of 3 independent biological experiments (n = 3) are presented. Two-way ANOVA followed by Dunnett’s multiple comparisons were performed to analyze statistically significant differences between the first data point and other data points within a treatment. Only the first significant difference is presented to avoid overcrowding of the figure; **** represents *P* < 0.0001.

**Schematic overview of the DFM setup**


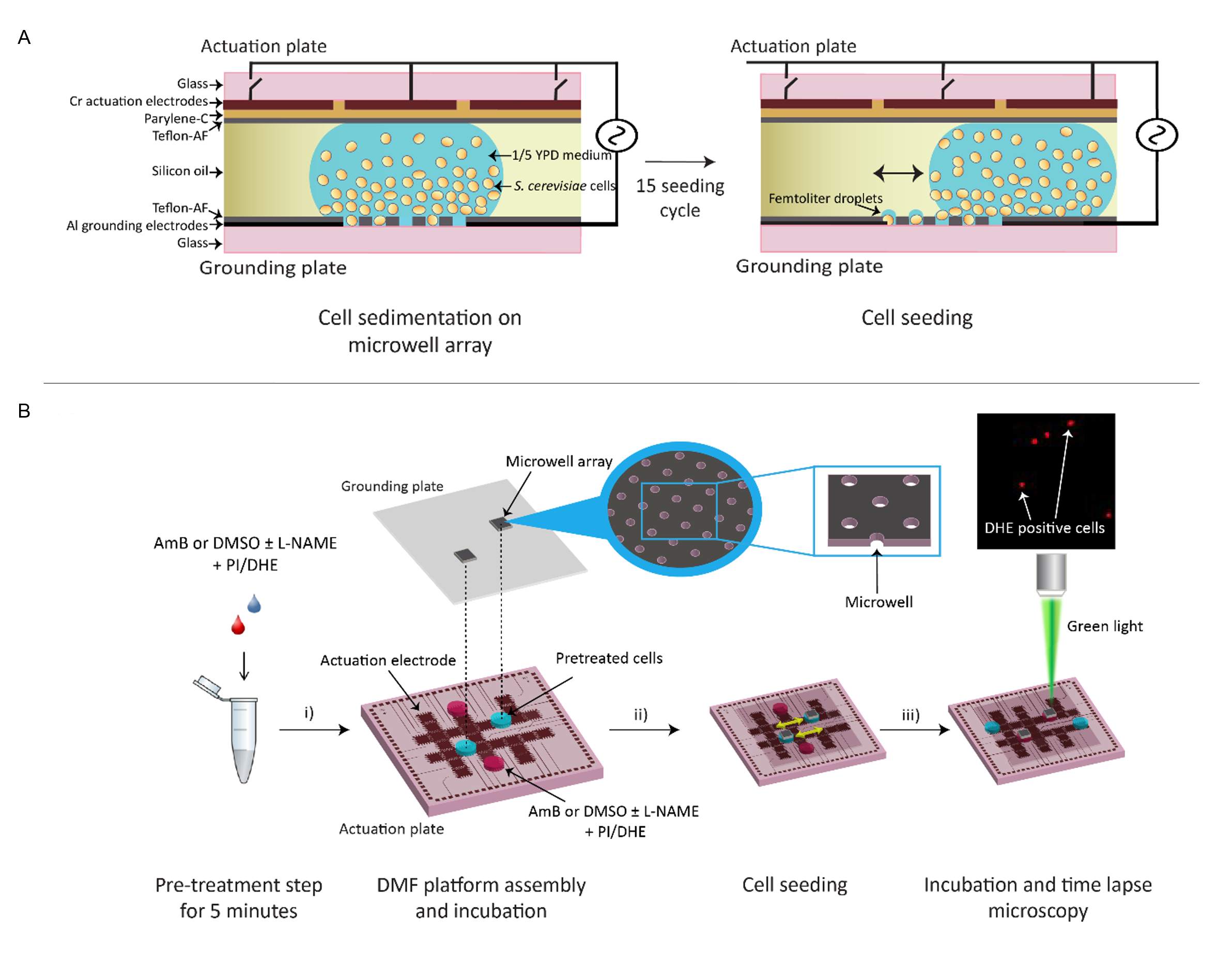


**S3 Fig. Schematic overview of the DMF setup.** (**A**) Grounding plate with microwell arrays used to seed single cells. The grounding plate contains wells to trap single cells by manipulating the droplet across the microwell array using EWOD. Cells are forced inside the wells due to the receding meniscus of the droplet and by hydrophobic/hydrophilic interactions between the Teflon layer and the glass substrate. **(B)** Actuation plate with electrodes to manipulate the droplets using EWOD representing subsequent steps to perform cytotoxicity assays at single cell level. Cells are mixed with treatment (DMSO, AmB, L-NAME or a combination) and stain (DHE or PI) and pre-incubated for 5 min off-chip. (i) Cell suspension droplets (blue) and treatment-stain droplets (red) are placed on the actuation plate and the actuation plate is covered with the grounding plates. Oil is added between the plates to prevent droplet evaporation and the DMF chip is placed in the chipholder. (ii) The cell suspension droplets are manipulated across the array using EWOD to seed single cells in the wells and after seeding, the cell suspension droplets are moved away and the treatment-stain droplets are manipulated to cover the microwell array. (iii) Cells are incubated for 3 h and subjected to time-lapse microscopy to monitor their fluorescence every 15 min.

**L-NAME does not significantly alter the killing potential of peroxide in *S. cerevisiae*.**

**S4 Fig. L-NAME does not significantly alter the killing potential of peroxide in *S. cerevisiae*.** Exponential yeast cells were treated with different concentrations of H_2_O_2_ in the absence (black bars) or presence (white bars) of 200 nM L-NAME for 180 min. Numbers of CFU/mL in Log-scale, assessed by plating assays and CFU counting are shown. Means and standard errors of the means (SEMs) of 3 independent biological experiments (n = 3) are presented. Two-way ANOVA followed by Tukey multiple comparisons test was performed to analyse significant differences between the two treatments.

**Time lapse experiments via bulk analysis in *S. cerevisiae* corroborate the results of the single cell analysis via the DMF platform.**

**A B C**

**S5 Fig. Time lapse experiments via bulk analysis in *S. cerevisiae* corroborate the results of the single cell analysis via the DMF platform.** Exponential yeast cells were treated with 10 μM AmB in the absence (black bars) or presence (white bars) of 200 mM L-NAME. Cells were analysed for their DHE- and PI-fluorescence in the bulk setup with FACS (A and B). Bulk plating assays were performed (C). Means and standard errors of the means (SEMs) of 2 independent biological experiments (n = 2) are presented. Two-way ANOVA followed by Tukey multiple comparisons test was performed to analyse significant differences between the two treatments; **, *** and **** represent *P* < 0.01, *P* < 0.001 and *P* < 0.0001, respectively.

**L-NAME acts fungistatic on *S. cerevisiae***

**S6 Fig. L-NAME acts fungistatic on *S. cerevisiae*.** Yeast cells were treated with different concentrations of L-NAME. The optical density of the culture relative to the control (no L-NAME) is shown. Means and standard errors of the means (SEMs) of 4 independent biological experiments (n = 4) are presented.
